# Comparative in situ analyses of cell wall matrix polysaccharide dynamics in developing rice and wheat grain

**DOI:** 10.1007/s00425-014-2201-4

**Published:** 2014-11-22

**Authors:** Richard Palmer, Valérie Cornuault, Susan E. Marcus, J. Paul Knox, Peter R. Shewry, Paola Tosi

**Affiliations:** 1 Rothamsted Research, Harpenden, AL5 2JQ UK; 2 Centre for Plant Sciences, Faculty of Biological Sciences, University of Leeds, Leeds, LS2 9JT UK; 3 School of Agriculture Policy and Development, University of Reading, Reading, RG6 6AH UK

**Keywords:** Cell wall, Grain development, Immunodetection, Rice grain, Wheat grain

## Abstract

Cell wall polysaccharides of wheat and rice endosperm are an important source of dietary fibre. Monoclonal antibodies specific to cell wall polysaccharides were used to determine polysaccharide dynamics during the development of both wheat and rice grain. Wheat and rice grain present near synchronous developmental processes and significantly different endosperm cell wall compositions, allowing the localisation of these polysaccharides to be related to developmental changes. Arabinoxylan (AX) and mixed-linkage glucan (MLG) have analogous cellular locations in both species, with deposition of AX and MLG coinciding with the start of grain filling. A glucuronoxylan (GUX) epitope was detected in rice, but not wheat endosperm cell walls. Callose has been reported to be associated with the formation of cell wall outgrowths during endosperm cellularisation and xyloglucan is here shown to be a component of these anticlinal extensions, occurring transiently in both species. Pectic homogalacturonan (HG) was abundant in cell walls of maternal tissues of wheat and rice grain, but only detected in endosperm cell walls of rice in an unesterified HG form. A rhamnogalacturonan-I (RG-I) backbone epitope was observed to be temporally regulated in both species, detected in endosperm cell walls from 12 DAA in rice and 20 DAA in wheat grain. Detection of the LM5 galactan epitope showed a clear distinction between wheat and rice, being detected at the earliest stages of development in rice endosperm cell walls, but not detected in wheat endosperm cell walls, only in maternal tissues. In contrast, the LM6 arabinan epitope was detected in both species around 8 DAA and was transient in wheat grain, but persisted in rice until maturity.

**Electronic supplementary material:**

The online version of this article (doi:10.1007/s00425-014-2201-4) contains supplementary material, which is available to authorized users.

## Introduction

Wheat and rice are the staple crops for up to two-thirds of the world’s population, providing more than 50 % of the daily calorific intake to nearly three billion people (http://faostat.fao.org/site/368/default.aspx). Cereals also contribute up to 50 % of the total dietary fibre in western diets (Bates et al. 2011). The major components of cereal grain fibre are cell wall polysaccharides, which account for ~2 % of the dry weight of white wheat flour or polished rice grain, but up to 20 % of whole grain (Juliano 1985). An understanding of the structures of cereal grain cell walls is, therefore, of direct relevance to the understanding of the role of cereals in human health. The benefits of increased dietary fibre intake include improved regulation of blood sugar, reduction in serum cholesterol, immune stimulation and decreased risk of some types of cancer (Bingham et al. 1985; Cade et al. 2007; Anderson et al. 2009; Bjorck et al. 2012; Threapleton et al. 2013). By contrast, lower contents of dietary fibre are required for other applications with the high viscosity resulting from soluble fibre being detrimental when cereals are used as feed for monogastric livestock such as pigs and poultry (Hesselman et al. 1981) and for the production of ethanol in brewing, distilling and biofuel plants.

The mature cereal grain is a single-seeded fruit (caryopsis) comprising the embryo and endosperm (which are derived from fertilisation events) surrounded by the pericarp and testa, (which are outer layers of maternal origin). Events during grain development can be grouped into four main stages: early development (including fertilisation and cellularisation), differentiation (including the formation of all major cell types), grain filling, and maturation/desiccation (Sabelli and Larkins 2009). Cellularisation of the future endosperm begins ~2 days after anthesis (DAA) with the formation of cell walls transforming a multinucleate cytoplasm into a multicellular structure. This process continues until, by 4–6 DAA, the entire cavity has been filled with cells (Mares et al. 1975; Brown et al. 1997; Sabelli and Larkins 2009). Subsequently, three types of endosperm cell are differentiated: central starchy endosperm cells, which comprise most of the tissue; the outer aleurone cell layer, which in wheat and most cultivated rice consists of a single layer of cells surrounding the endosperm and containing oil bodies and protein bodies; and the sub-aleurone cells, which comprises two to three layers of cells immediately below the aleurone. These cell types are clearly differentiated by 12 DAA, although division of the aleurone cells may continue for several more days, giving rise to the sub-aleurone cells (Evers 1970; Cochrane and Duffus 1981). Grain filling begins at about 10-12 DAA, but is most active between about 14 and 28 DAA, resulting in the deposition of storage polymers, predominantly starch but also storage proteins, in the central starchy endosperm cells, with the sub-aleurone cells being particularly rich in protein (Toole et al. 2009; He et al. 2013). Finally, after about 28 DAA, the grains undergo maturation with desiccation resulting in a water content of about 15 % dry weight.

Plant cell walls are composites of polysaccharides, with cellulose as a fibrous component and sets of matrix polysaccharides. These matrix polysaccharides include glucans, heteroxylans, heteromannans (often referred to as hemicelluloses) and pectic polysaccharides which are often present in supramolecules containing a range of pectic domains (Burton et al. 2010). The endosperm cell walls of the grasses typically have low levels of cellulose, xyloglucan and pectins and high contents of arabinoxylan (AX) and mixed-linkage glucan (MLG) relative to the cell walls of non-graminaceous plants, although the relative amounts of AX and MLG can vary substantially between cereal species and different grain tissues. Thus, AX comprises ~20 % total cell wall polysaccharides of the starchy endosperm in barley, 25 % in rice and 70 % in wheat whilst MLG accounts for over 70 % in barley and ~20 % in the other two species. However, rice has significantly higher levels of cellulose (23 % compared with 2 % in wheat and 3–4 % in barley) and about 27 % pectin, which is not significant in wheat or barley grain (Mares and Stone 1973; Shibuya et al. 1983, 1985; Shibuya and Nakane 1984; Shibuya 1989). Wheat endosperm cell walls also contain ~7 % glucomannan (Mares and Stone 1973) compared to 3–4 % in barley whilst the presence of low levels of xyloglucan has been shown by immunolabelling in both these cereals (Pellny et al. 2012; Wilson et al. 2012).

Cereal AX has a backbone of xylose residues that can be mono-substituted with arabinose residues at the O-3 or di-substituted at the O-2 and O-3 positions. FT-IR and Raman spectroscopic analyses have shown that the degree of substitution differs between developmental stages and between cells at different positions within the endosperm (Toole et al. 2010). The variation in substitution level between mono- and di-substituted AX is thought to regulate the hydration status of the cell wall, affecting its flexibility and potentially the nutrient transfer rate (Toole et al. 2011). The AX of grasses is typically esterified with ferulic acid at the five position of arabinose residues and this is thought to provide extra structural strength in the cell wall matrix through the ability to form ether linkages between ferulic residues present on adjacent AX chains (Piot et al. 2001). In general, grass MLG contains single 1,3-β-glucan linkages interspersed by three or four, 1,4-β-glucan linkages (Burton and Fincher 2009) with continuous stretches of up to fourteen 1,4 linkages being reported in wheat bran although these are a minor component (Cui and Wood 2000). The ratio and distribution of these two types of linkage may have profound effects on the structural characteristics including the ability to form inter-chain interactions (Lazaridou and Biliaderis 2007). Callose (1,3-β-glucan) has also been demonstrated to be an essential component of the first anticlinal cell wall extensions during cellularisation (Morrison and Obrien 1976; Fineran et al. 1982; Wilson et al. 2006) and early cell wall development (cell plate deposition) in wheat, rice, barley and other species (Verma and Hong 2001; Philippe et al. 2006b; Wilson et al. 2012) where it appears as a transient component during cellularisation. As already mentioned, pectin is a very minor component of endosperm cell wall of wheat and barley, but a substantial component of cell wall polysaccharides in rice endosperm. Pectic polysaccharides all contain 4-linked galacturonic acid (GalA) residues and can be classified into three main types: homogalacturonan (HG), rhamnogalacturonan-I (RG-I), and rhamnogalacturonan-II (RG-II). These three types of pectin are important polymers of the cell wall matrix (Caffall and Mohnen 2009) and are proposed to be covalently linked to one another to form supramolecules, the structures of which are still poorly understood.

As discussed above, gradients in the amounts of some cell wall polysaccharides across the wheat endosperm have been identified, but little is known about the factors controlling these gradients or their biological roles and not all cell wall matrix polysaccharides have been studied. It is possible that these gradients are related to cell age and lineage, since the sub-aleurone layer is thought to derive from periclinal cell divisions of aleurone cells, occurring later into grain development than the divisions of central endosperm cells that give rise to the central starchy endosperm (Olsen et al. 1998; Olsen 2001). Although the formation of cell walls in the developing rice endosperm is well described and the polysaccharide composition of the mature grain identified, the sequence of deposition of individual wall polysaccharides has not been reported. Wheat and rice grain present important anatomical differences, first of all, the presence of a crease in wheat accommodating the vascular bundle and acting as the sole point of entry of assimilates in the endosperm; in rice, on the contrary, nutrients are unloaded from the phloem in the nucellar epidermis, can move circumferentially and enter the endosperm at different points via the aleurone cells. Cell wall composition and formation dynamics in the two species may, therefore, reflect this different grain physiology. The aim of the present study was, therefore, to perform a comparative analysis and determine the temporal and spatial patterns of polymer deposition in cell walls of developing rice grain, focusing on the endosperm, and to compare these with the pattern in wheat, which has been more thoroughly described. This was achieved using immunofluorescence microscopy with sets of monoclonal antibodies (mAbs) to detect the cell wall matrix polysaccharides, focusing on three major time points selected to represent key stages of grain development in both species.

## Materials and methods

### Plant material

*Oryza sativa* cv. Koshihikari (bred at Fukui Prefectural Agricultural Research Facility) plants were grown in 15-cm diameter pots under controlled environment conditions at Rothamsted Research with 12-h light period at 28 °C daytime temperature and 22 °C nighttime temperature, 70 % relative humidity. Pots were placed in simulated paddy field conditions, where the pots are two-thirds submerged in a deep tray of water. Seeds were germinated in dark moist conditions and transferred to hydroponic conditions after 7 days. Seedlings were subsequently transferred to loam-based soil once they had reached a height of 15 cm. Caryopses were harvested at 4, 6, 8, 12, 20 and 28 DAA from the middle third of the panicle and immediately prepared for microscopy. Anthesis was defined as the point at which the middle third of the panicle had exposed anthers. *Triticum aestivum* cv. Cadenza (bred by Cambridge Plant Breeders Ltd.) plants were grown under glasshouse conditions at Rothamsted Research, as previously described (Tosi et al. 2004). Caryopses were harvested at 4, 6, 8, 12, 20 and 28 DAA from the middle third of the spikelet and immediately prepared for microscopy.

### Light microscopy and immunofluorescence analysis

Transverse medial sections of wheat and rice grains (approximately 1 mm in thickness) were cut in fixative. Sections were fixed overnight at room temperature (RT) in 4 % (w/v) paraformaldehyde and 2.5 % (w/v) glutaraldehyde in 0.1 M Sorenson’s phosphate buffer. After three rinses in buffer, the specimens were dehydrated in an ethanol series, slowly infiltrated with LR White resin (25, 50, 75, 100 %, (v/v); medium grade, TAAB L012) for 7 and 28 days for rice and polymerised at 55 °C in a nitrogen gas saturated environment. Semi-thin sections of 1 μm thickness were cut using a Reichert–Jung ultramicrotome, collected in drops of distilled water on multi-well slides coated with poly-l-lysine hydrobromide (Sigma P1399), and dried on a hot plate at 40 °C.

Slides with LR White-embedded grain sections were pre-incubated (50 μl drop/well) in 5 % (w/v) milk powder (Marvel products) in 1xPBS at pH 7.0 for 60 min, then incubated for 2 h in primary antibody. The following monoclonal antibodies were used, diluted in PBS containing 5 % (w/v) milk powder: rat monoclonal—LM5 (Jones et al. 1997), LM6 (Willats et al. 1998), LM19 (Verhertbruggen et al. 2009), LM25 (Pedersen et al. 2012), JIM7 (Knox et al. 1990) all diluted 1:5; mouse monoclonal AX1 (Guillon et al. 2004), anti-callose (Meikle et al. 1991) (BioSupplies Australia, Cat No. 400-2), anti- MLG (Meikle et al. 1994) (BioSupplies Australia, Cat No. 400-3) diluted 1:50; mouse monoclonal INRA-RU1, (Ralet et al. 2010) (INRA Nantes) diluted 1:5. Slides were rinsed three times for 5 min with 1xPBS, then incubated for 2 h, in the dark, with secondary antibody (anti-rat Alexa 568 conjugated or anti-mouse Alexa 568 conjugated, Invitrogen) diluted 1:200 in PBS, 5 % (w/v) milk powder. Slides were then rinsed three times with 1xPBS, and counterstained with 1 % (w/v) Calcofluor White solution. Sections were then mounted in Citifluor AF1 glycerol-based antifade mountant and analysed on a Zeiss Axiophot fluorescence microscope equipped with a Retiga Exi (Qimaging) camera.

## Results

The in situ location of cell wall matrix polysaccharides was compared in transverse sections (TS) of developing grain of wheat and rice and focused on three key developmental stages: 4 DAA (cellularisation), 12 DAA (end of cell differentiation, start of grain filling) and 28 DAA (end of grain filling, start of maturation). The monoclonal antibodies directed to cell wall matrix polysaccharides used for these analyses are listed in Table 1.

**Table 1:** Cell wall directed monoclonal antibodies used in this study

Antibody	Antigen	References
INRA-AX1	Arabinoxylan	Guillon et al. (2004)
LM28	Glucuronoxylan	Cornuault, Marcus, Knox (data not shown)
MLG	Mixed link beta glucan	Meikle et al. (1994)
Callose	1-3 β-glucan	Meikle et al. (1991)
LM21	Heteromannan	Marcus et al. (2010)
LM25	Xyloglucan	Pedersen et al. (2012)
LM19	Unesterified homogalacturonan	Verhertbruggen et al. (2009)
JIM7	Methyl-esterified pectic HG	Clausen et al. (2003)
INRA-RU1	Rhamnogalacturonan backbone	Ralet et al. (2010)
LM5	(1-4)-β-d-galactan	Jones et al. (1997)
LM6	(1-5)-α-l-arabinan	Willats et al. (1998)

**Fig. 1:**
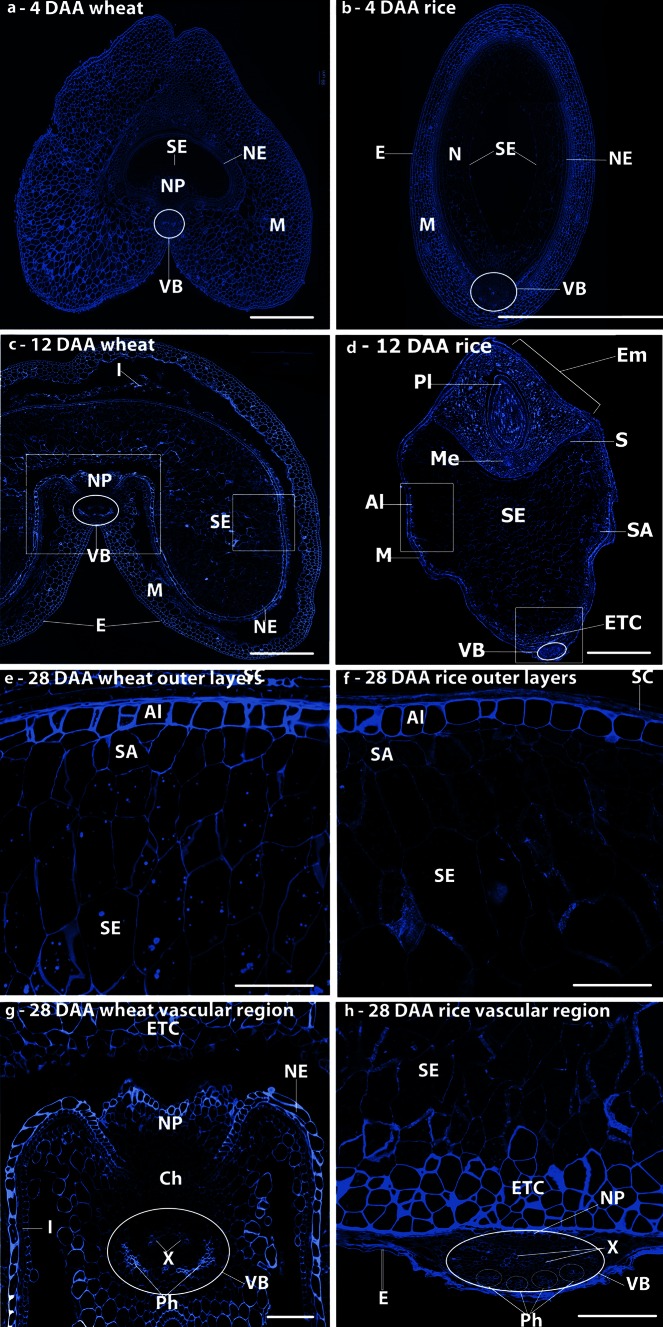
Histochemical labelling of transverse sections with Calcofluor White 2MR of wheat grains (**a**, **c**, **e**, **g**) and rice grains (**b**, **d**, **f**, **h**) at 4 (**a**, **b**), 12 (**c**, **d**), and 28 DAA (**e**, **f**), and enlargements of the crease regions (**g**, **h**) showing the cell structures in the two grains. *M* maternal pericarp, *NP* nucellar projection, *Ph* phloem, *X* xylem, *NE* nucellar epidermis, *N* nucellus, *SA* sub-aleurone, *Al* aleurone, *ETC* endosperm transfer cells, *Pl* plumule, *Me* mesocotyl, *S* scutellum, *SE* starchy endosperm, *Ch* chalazal region, *E* epidermis, *VB* vascular bundle. *Bars* 500 μm (**a**–**d**), *bars* 100 μm (**e**–**h**)

The anatomy of the grain sections was studied using Calcofluor White, which stains β1-3 and β1-4 polysaccharides, including cellulose, callose and MLG (Fig. 1). Wheat and rice share a similar grain development pattern and are synchronous in development when grown under standard conditions. However, there are significant differences in the grain structure of these two cereals, with wheat developing a characteristic crease region running the length of the grain. The crease is centred upon a single vascular bundle (VB in Fig. 1a, c) that provides the nutrition required by the developing embryo and endosperm via the nucellar projection (NP in Fig. 1a) and endosperm cavity (Wang et al. 1993, 1994). By contrast, in rice grain the endosperm remains ovoid in cross-section with the vascular bundle located on the dorsal side of the grain (VB in Fig. 1b, d). Consequently, whereas the nutrients are transported radially into the endosperm from the vascular bundle in wheat, they pass around the outer tissues of the rice caryopsis before being transported into the endosperm in rice (Oparka and Gates 1981a, b, 1984). At 4 DAA, the endosperm of both species (SE in Fig. 1a, b) is undergoing cellularisation and is surrounded by extensive layers of maternal cells (mainly pericarp) (M in Fig. 1a, b). By 12 DAA (Fig. 1c, d), both caryopses have expanded, although the expansion is greater for wheat than for rice, with clearly defined starchy endosperm, sub-aleurone and aleurone tissues within the endosperm. By 28 days, both species have expanded greatly. The outer endosperm regions are shown in Fig. 1e, f, the groove region of wheat in Fig. 1g and a vascular bundle and the adjacent endosperm region of rice in Fig. 1h.

### Dynamics of non-cellulosic/non-pectic cell wall matrix glycans

#### Arabinoxylan (AX)

**Fig. 2:**
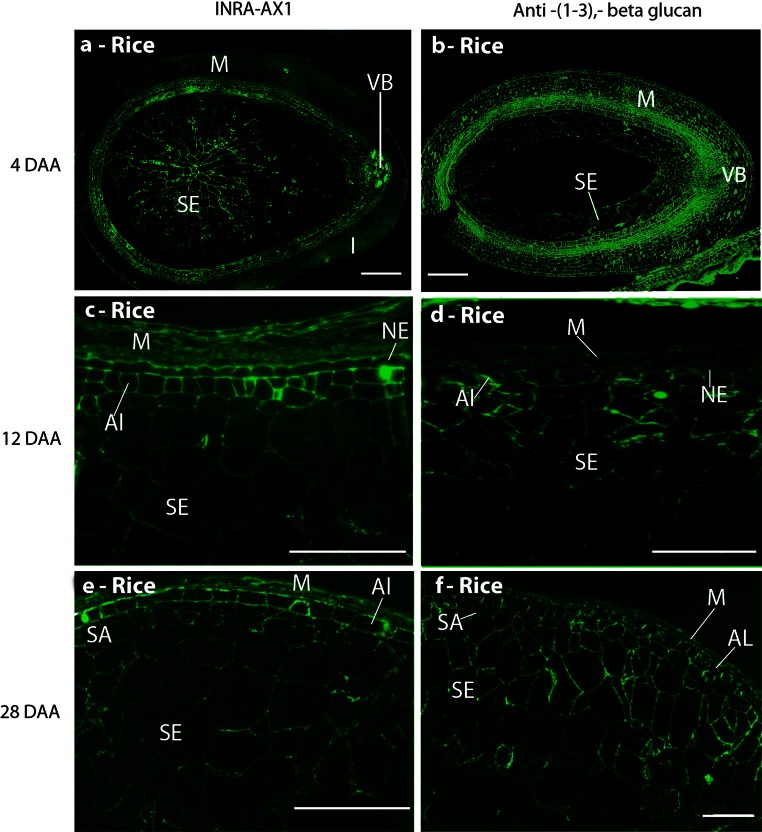
Indirect immunofluorescence detection of AX and callose in medial transverse sections of a rice grain at 4 (**b**), 6 (**a**), 12 (**c**, **d**), and 28 DAA (**e**, **f**). Immunofluorescence detection of AX (**a**, **c**, **e**) and callose (**b**, **d**, **f**). *M* maternal pericarp, *NP* nucellar projection, *NE* nucellar epidermis, *SA* sub-aleurone, *Al* aleurone, *SE* starchy endosperm, *VB* vascular bundle. *Bar* 100 μm

The INRA-AX1 monoclonal antibody was used to detect AX. The patterns of AX epitope detection in developing wheat grain have been studied extensively (Guillon et al. 2004; McCartney et al. 2005; Philippe et al. 2006a; Dornez et al. 2011; Robert et al. 2011; Pellny et al. 2012). In the current study, the INRA-AX1 epitope was detected in the cell walls of the nucellar epidermis and nucellar projection closest to the endosperm tissue at 8 DAA, whilst the modified aleurone cells in the groove region labelled strongly at 12 DAA, with weaker labelling extending radially across the endosperm towards the outer layer of cells which are differentiated from the aleurone (Fig. S1 d). The intensity of labelling increased towards maturity (28 DAA), when all of the endosperm cells were clearly labelled (Fig. S1 g). By contrast, in rice, labelling with INRA-AX1 was observed in all the central endosperm cells from as early as 6 DAA and increased in intensity throughout grain development. In the aleurone cells, the labelling was particularly strong from 16 DAA, after they had differentiated cell wall thickenings (Fig. 2e). In addition, a recently isolated MAb, LM28 (Cornuault V, Marcus SE, Knox JP, Faculty of Biological Sciences, University of Leeds, UK, data not shown) binding to a glucuronosyl-containing epitope widely present in heteroxylans, was used for immunolocalisation of glucuronoxylan (GUX).

**Fig. 3:**
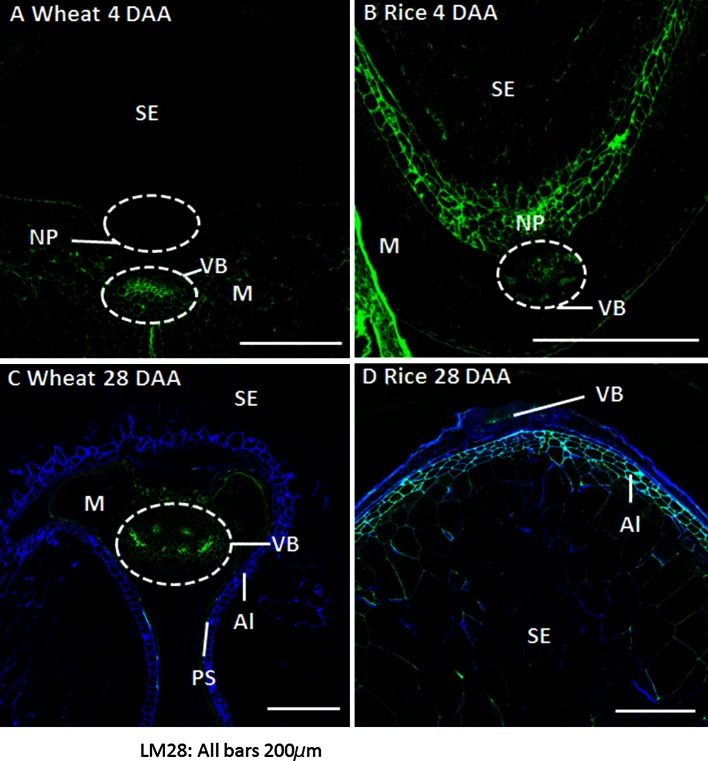
Indirect immunofluorescence detection of glucuronoxylan (GUX, LM28) in medial transverse sections of wheat (**a**, **c**) and rice (**b**, **d**) grains at 4 DAA (**a**, **b**) and 28 DAA (**c**, **d**). *M* maternal pericarp, *NP* nucellar projection, *Al* aleurone, *PS* pigment strand, *SE* starchy endosperm, *VB* vascular bundle, *H* husk. *Bar* 200 μm

In wheat, detection of the GUX epitope was restricted to the maternal tissues at all stages of grain development analysed, with stronger labelling observed on cell walls of the epidermis and of phloem and xylem vessels (Fig. 3a, c). From 20 DAA onwards, the epitope was also abundant in residual cell walls of the nucellar epidermis proximal to the aleurone tissues. Conversely, in rice grains, the GUX epitope appeared widespread throughout both maternal and endosperm tissues from just after the completion of cellularisation, at 4 DAA, until 28 DAA (Fig. 3b, d). The labelling of the endosperm by the LM28 at 4DAA appears even, encompassing all endosperm cell walls (Fig. 3b), but by 8 DAA has already become more weak and irregular. By 28 DAA strong labelling is detected only in aleurone and outer starchy endosperm cells whilst cells in the central region show little or no labelling (Fig. 3d). The aleurone cells can be differentiated from the endosperm cells from 8 DAA with the presence of strong detection of the GUX epitope which persists until maturity.

#### Callose

Callose (1,3-β-glucan) was detected in the extending anticlinal cell walls of both rice (Fig. 2b) and wheat (Fig. S1 b) at cellularisation, and continued to be observed throughout the endosperm at all later time points. Stronger labelling of the putative aleurone and sub-aleurone cells was also observed, compared with weaker punctate labelling of the central starchy endosperm cell walls (Fig. 2d, f and S1 e, h). These results are consistent with previous studies (Morrison and Obrien 1976; Fineran et al. 1982; Brown et al. 1997; Li et al. 2003).

#### Mixed-linkage glucan

**Fig. 4:**
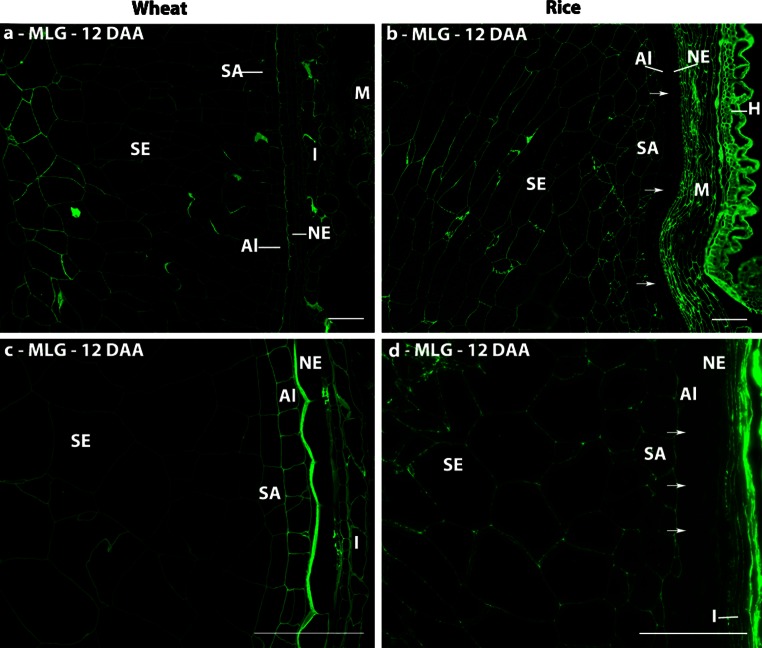
Indirect immunofluorescence detection of MLG in medial transverse sections of wheat (**a**, **c**) and rice (**b**, **d**) grains at 12 DAA. *Arrowheads* indicate absence of fluorescence labelling in the aleurone and nucellar epidermis. *M* maternal pericarp, *NP* nucellar projection, *NE* nucellar epidermis, *SA* sub-aleurone, *Al* aleurone, *I* Integuments, *SE* starchy endosperm, *VB* vascular bundle, *H* husk. *Bar* 100 μm

Detection of MLG was restricted to the maternal tissues of both species at the cellularisation stage, where it was detected in phloem vessels, the nucellar epidermis and integuments. The endosperm tissues of both species were labelled with the MLG antibody by 8 DAA and remained so throughout development. However, clear differences between the two species were observed at 12 DAA (Fig. 4). In wheat, the nucellar epidermis and aleurone cell walls are clearly labelled, but the labelling is weaker in the cells below the immediate sub-aleurone layer (which are thought to be derived from recent divisions of aleurone cells and hence retain aleurone characteristics) than in the central starchy endosperm cells (Fig. 4a, c). This pattern is consistent with the previous study of Philippe et al. (2006a). By contrast, the vascular bundle, nucellar epidermis and aleurone cell walls of rice are not labelled, but labelling of the sub-aleurone and starchy endosperm cells is more even in intensity than in wheat (Fig. 4b, d). However, the aleurone cell walls of both species were strongly labelled by 20 DAA (data not shown) and remained so until maturity.

#### Xyloglucan

**Fig. 5:**
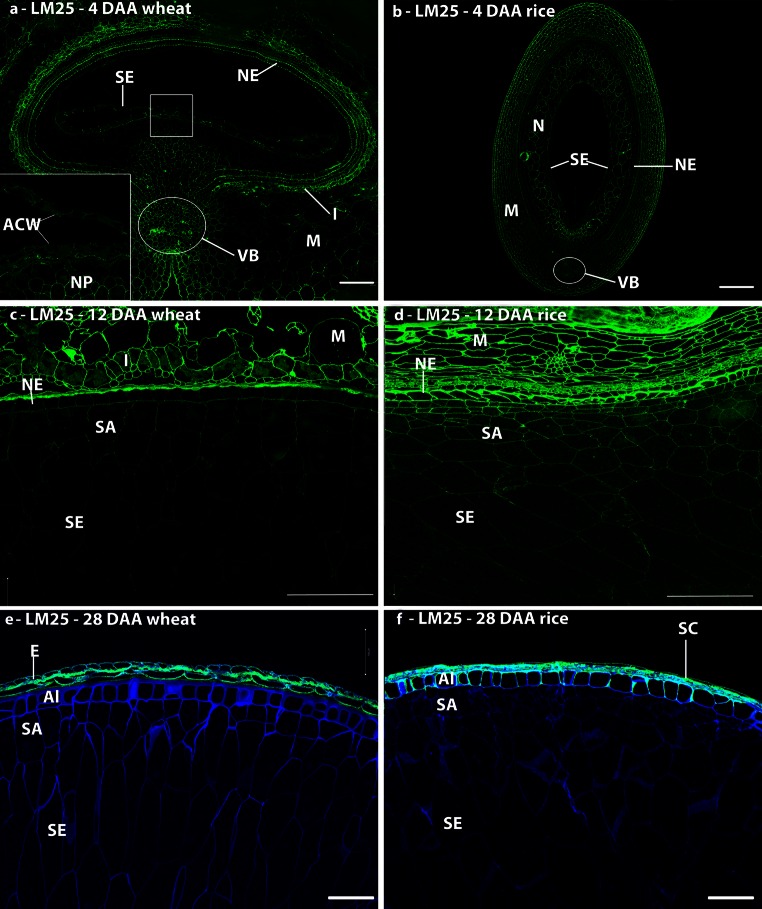
Indirect immunofluorescence detection of xyloglucan in medial transverse sections of wheat (**a**, **c**, **e**) and rice (**b**, **d**, **f**) grains at 4 (**a**, **b**), 12 (**c**, **d**), and 28 days after anthesis **(e**, **f**). *Inset* in micrograph **a** is a ×4 enlargement of the *boxed region*, showing immunofluorescence labelling of the anticlinal cell walls. *M* maternal pericarp, *N* nucellus, *NP* nucellar projection, *NE* nucellar epidermis, *SA* sub-aleurone, *Al* aleurone, *I* integuments, *SE* starchy endosperm, *VB* vascular bundle, *ACW* anticlinal cell wall. *Bar* 100 μm

Xyloglucan has not been identified in the cell walls of wheat endosperm by biochemical analyses, but its presence in early stages of development has been shown using the LM15 antibody (Pellny et al. 2012). Studies with the more recently generated LM25 xyloglucan antibody confirmed the presence of xyloglucan in developing grain with abundant detection in the cell walls undergoing cellularisation in the syncytial endosperm of both species (Fig. 5a, b), but reduced/loss of labelling after 12 DAA (Fig. 5c–f). Labelling of the aleurone cell walls persisted until 28 DAA in rice (Fig. 5f), but was lost by this stage in wheat (Fig. 5e).

#### Glucomannan

**Fig. 6:**
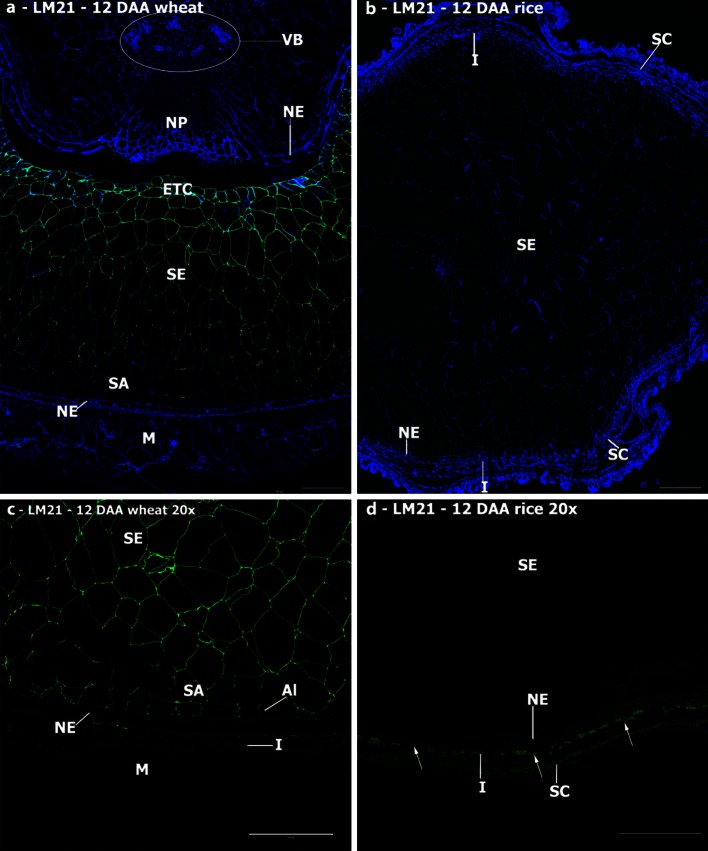
Indirect immunofluorescence detection of heteromannan in medial transverse sections of wheat (**a, c**) and rice (**b, d**) grains at 12 DAA. Micrographs **c** and **d** are ×4 enlargements of the outer endosperm regions of micrographs **a** and **b** to show that the heteromannan signal is present throughout the endosperm and sub-aleurone tissues in wheat, but remains absent in rice. *M* maternal pericarp, *N* nucellus, *NP* nucellar projection, *NE* nucellar epidermis, *SA* sub-aleurone, *Al* aleurone, *I* integuments, *SE* starchy endosperm, *VB* vascular bundle, *ETC* endosperm transfer cells, *SC* seed coat. *Bar* 100 μm

Glucomannan has been identified in starchy endosperm and aleurone cell walls of wheat by biochemical analyses (Mares and Stone 1973), and recent analyses with the LM21 heteromannan antibody detected mannan epitopes throughout development (Pellny et al. 2012). Our studies confirm these results for wheat, with strong but uneven detection in starchy endosperm and sub-aleurone cell walls (Fig. 6a, c). In sections of wheat grain, the LM21 epitope was detected in aleurone cell walls weakly at 12 DAA (Fig. 6c) and not at maturity. By contrast, no detection of the LM21 epitope in endosperm cell walls of rice was observed at any developmental stage, although it was observed in the outer maternal tissues (Fig. 6b, d).

### Dynamics of pectic polysaccharides

#### Homogalacturonan

The potential presence of glycan domains of pectic supramolecules was studied using sets of antibody probes directed to HG and RG-I polysaccharides.

**Fig. 7:**
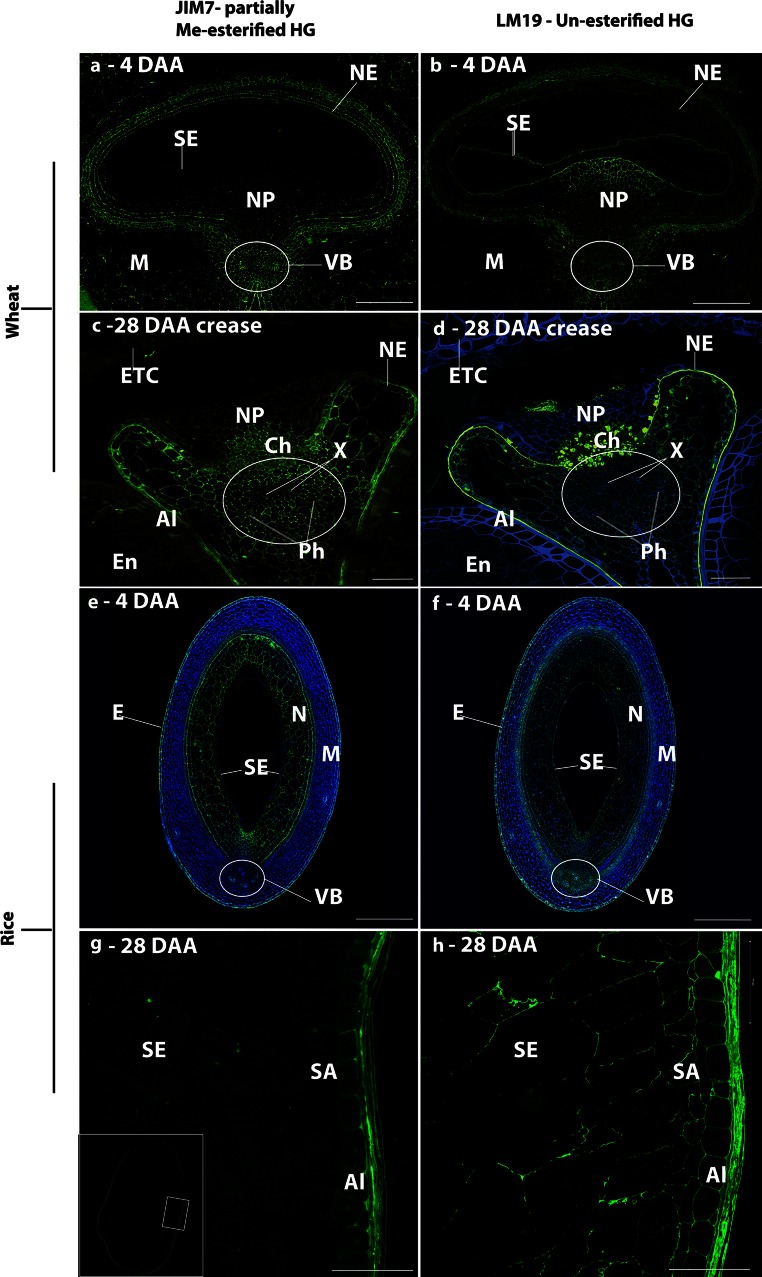
Indirect immunofluorescence detection of pectic HG in medial transverse sections of wheat (**a**–**d**) and rice (**e**–**h**) grains at 4 (**a**, **b**, **e**, **f**) and 28 DAA (**c**, **d**, **g**, **h**) using JIM7 and LM19 monoclonal antibodies. *Inset* in micrograph **g** is a lower magnification image with the *boxed region* indicating the region imaged for micrographs **g** and **h**. *M* maternal pericarp, *N* nucellus, *NP* nucellar projection, *Ph* phloem, *X* xylem, *NE* nucellar epidermis, *SA* sub-aleurone, *Al* aleurone, *I* integuments, *SE* starchy endosperm, *VB* vascular bundle, *E* epidermis, *Ch* chalazal region, *ETC* endosperm transfer cells. *Bar* 100 μm

The JIM7 antibody specific for methyl-esterified HG showed that esterified HG was present in the maternal tissues of wheat (Fig. 7), in particular the crease region, throughout development, with no labelling of the endosperm tissues. The patterns of labelling with LM19 (specific for unesterified HG) differed from those observed with JIM7. In particular, it was restricted to the cells of the nucellar projection closest to the endosperm cavity in the early stages of development (4–12 DAA) of wheat (Fig. 7b), but the remnants of these cells labelled only weakly after 12 DAA (Fig. 7d). Strong labelling with LM19 was also observed in the walls of the nucellar epidermis of wheat at 12 DAA, which persisted until 28 DAA (Fig. 7d). By contrast, a wider pattern of labelling with JIM7 and LM19 was observed in rice, including the cell walls of the endosperm, aleurone and maternal tissues (Fig. 7f, h). In the endosperm of rice grain, the LM19 epitope was the major epitope detected indicating the presence of unesterified HG.

#### Rhamnogalacturonan-I

**Fig. 8:**
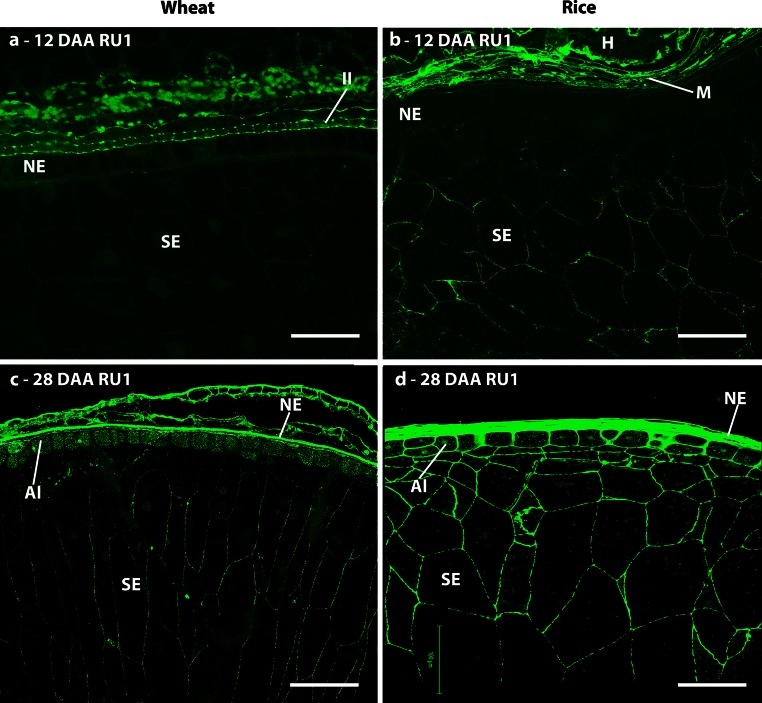
Indirect immunofluorescence detection of RG-I back bone in medial transverse sections of wheat (**a**, **c**) and rice (**b**, **d**) grains at 12 (**a**, **b**) and 28 days after anthesis (**c**, **d**). *M* maternal pericarp, *NE* nucellar epidermis, *SA* sub-aleurone, *Al* aleurone, *I* integuments, *SE* starchy endosperm, *II* inner integuments, *H* husk. *Bar* 100 μm

The presence of RG-I was determined using the INRA-RU1 antibody specific for the RG-I backbone and two antibodies specific for RG-I side chains: LM5 for 1,4-galactan and LM6 for 1,5-arabinan. Weak detection of the INRA-RU1 epitope was observed in the cell walls of the central starchy endosperm of wheat from 20 DAA, but not in those of the sub-aleurone or aleurone cells. In rice, the same pattern of labelling was observed from 12 DAA, but by 28 DAA the labelling also included the sub-aleurone and aleurone cells (Fig. 8b, d). Labelling of all maternal tissues, and in particular of the vascular regions, was considerably stronger at all time points in both species, which is consistent with the reported presence of significant amounts of pectins in these tissues (Shibuya et al. 1985; Hay and Spanswick 2006). In wheat, the RU1 epitope was localised at the triangular cell wall junction zones in the maternal pericarp prior to 12 DAA after which these regions are crushed and become indistinguishable. In rice, however, the INRA-RU1 epitope was more widely distributed throughout the walls of all pericarp cells.

**Fig. 9:**
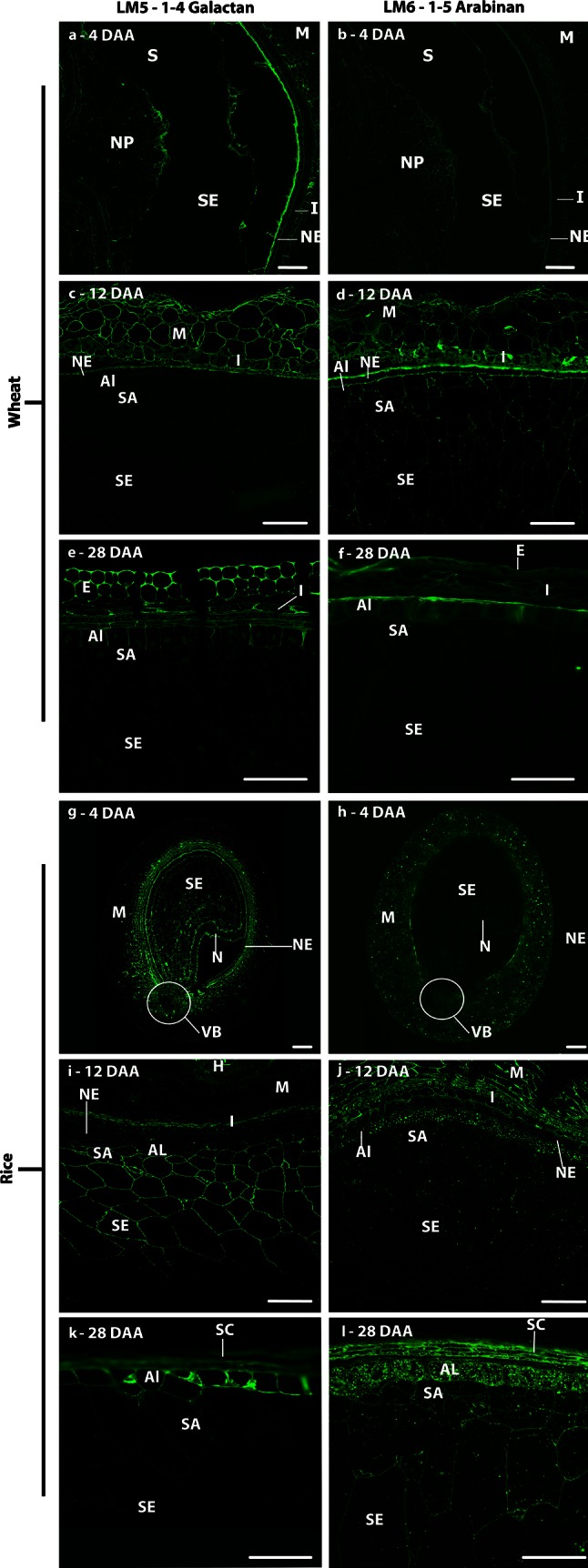
Indirect immunofluorescence detection of pectic arabinan and galactan as in medial transverse sections of wheat (**a**–**f**) and rice (**g**–**l**) grains at 4 (**a**, **b**, **g**, **h**), 12 (**c**, **d**, **I**, **j**) and 28 days after anthesis (**e**, **f**, **k**, **l**). *M* maternal pericarp, *NE* nucellar epidermis, *SA* sub-aleurone, *Al* aleurone, *I* integuments, *SE* starchy endosperm, *S* syncytium, *I* integuments, *SC* seed coat, *H* husk, *VB* vascular bundle, *N* nucellus, *NP* nucellar projection, *E* epidermis. *Bar* 100 μm

The endosperm cell walls of wheat were weakly labelled with LM6 by 8 DAA (data not shown) and were still labelled at 12DAA (Fig. 9d), but the epitope was not observed at 28 DAA (Fig. 9f). The only putative indication of RG-I in wheat endosperm, therefore, was the transient detection of arabinan at early developmental stages. This is in clear contrast to the ubiquitous detection of arabinan in the endosperm of rice at all stages after cellularisation. The LM5 and LM6 epitopes were weakly detected in the maternal tissues at all developmental stages in both species with the LM5 epitope being consistently more abundant in the inner pericarp tissues than in the outer pericarp tissues (Fig. 9a). However, the LM5 galactan epitope was not detected in wheat endosperm cells, but was detected in the cellularising endosperm of rice, with every endosperm cell being labelled at 4 DAA (Fig. 9g). After this, it became increasingly restricted to the outer layers of the endosperm: to the aleurone and sub-aleurone by 12 DAA (Fig. 9I) and to the aleurone layer only at 28 DAA (Fig. 9k). The LM6 arabinan epitope displayed a different pattern of distribution to the LM5 epitope in rice with little or no labelling at 4 DAA (Fig. 9h), but weak detection in all endosperm cells by 8 DAA. All starchy endosperm and sub-aleurone cells were labelled at 28 DAA, with the inner face of the aleurone cell walls being most strongly labelled (Fig. 9l). In these cells, it was also notable that the LM6 epitope occurred abundantly in intracellular structures in addition to the cell walls.

## Discussion

### Hemicellulose cell wall polysaccharides and grain development

The endosperm cell walls of wheat and rice grain are known to be rich in hemicelluloses, particularly AX and MLG, and to contain low levels of cellulose. Despite significant differences in the proportions of individual hemicelluloses, their patterns of deposition are both synchronous and spatially largely similar in wheat and rice. This suggests that they play an important role in the structure and mechanical properties of grain cell walls. Hemicelluloses are sets of polysaccharides that are proposed to cross-link to cellulose microfibrils to generate load-bearing structures (Scheller and Ulvskov 2010). However, the diversity of structures within the hemicellulose group and their varying dynamics are indicative of a range of specific roles in cell wall matrices during cell wall formation and development. The impacts of the occurrence of a particular polysaccharide whether heteroxylan, MLG, xyloglucan or heteromannan on cell wall properties are far from understood—not least in cell walls where cellulose levels are low, such as those in the grains of wheat and other cereals. The walls of the endosperm cells have several distinct roles during grain development. Firstly, they must be rapidly synthesised to accommodate grain expansion from 4 to 12 DAA. They must also be able to regulate cell hydration level and allow soluble assimilate exchange from cell to cell. Thirdly, they must show sufficient flexibility and strength to accommodate the mechanical stresses during grain expansion and subsequent desiccation. Finally, cell walls may play a role in seed dormancy and germination, months or even years, after desiccation (Finch-Savage and Leubner-Metzger 2006).

The high levels of hemicellulose polysaccharides in cereal endosperm cell walls may confer more plastic and adaptable properties than the cellulose microfibrils that are major components of most plant cell walls, allowing the cells to respond to the rapid changes that occur within a developing grain. Alternatively, they may represent a more easily digestible storage medium than microfibrillar cellulose. The deposition of AX in the endosperm cell walls of rice follows the well-characterised deposition of this polysaccharide in wheat (Philippe et al. 2006b) with its first detection by the LM11 antibody coinciding with the cessation of the most rapid phase of grain expansion, which occurs at about 12 DAA in wheat (Gao et al. 1992; Shewry et al. 2012) and 8 DAA in rice (Hoshikawa 1973). However, work carried out on developing barley grain has shown that pretreatment of sections with α-arabinofuranosidase allows detection of AX by LM11 from as early as 5 DAA (end of cellularisation), suggesting that AX is initially deposited as an heavily substituted form not recognised by this antibody (Wilson et al. 2012). It is plausible that a similar process takes place also in wheat and rice endosperm; a decrease in AX substitution level in the course of grain development (Toole et al. 2009) has been indeed reported for wheat. Glucuronosyl substitutions of AX are known to be present in pericarp and seed coat tissues of cereals (Fincher and Stone 2004). In this paper, we showed that whilst the LM28 GUX epitope is present in the maternal tissues of wheat throughout grain development, it was not detected at any stage in the cell walls of the endosperm. This was in contrast with what was observed in rice, where the LM28 antibody labelled both maternal and endosperm tissues, although with a different pattern and intensity of labelling in the different stages of development, suggesting a clear difference between the structure of AX in wheat and rice endosperm. Strong labelling by the LM28 antibody allows us to discriminate the rice aleurone cells from the rest of the endosperm cells from 8 DAA, significantly earlier than with other cell wall antibodies or microscopy stains.

This labelling is also significant as very little AX can be detected in these cells at this time point, perhaps indicating that glucuronosyl substitutions may interfere with AX detection with INRA AX-1. MLG is also deposited at early stages of development in both wheat and rice (by 6 DAA) with similar proportions being present at maturity in both species (~23 and 25 %, respectively). A difference was seen in MLG detection in the aleurone and nucellar epidermis: in rice MLG was not detected during early development, however, at 28 DAA the aleurone is heavily labelled with the anti-MLG antibody, whereas in wheat, similarly to barley (Wilson et al. 2006, 2012), these tissues exhibit clear labelling at all stages after 8 DAA. These differences in the deposition of MLG may function to regulate soluble assimilate exchange from the vascular bundle to the endosperm cells, reflecting differences in the way assimilate are delivered to the endosperm cells in the three cereal grains: solely via the nucellar projection in wheat and barley, whilst also circumferentially via the nucellar epidermis and through the aleurone cells in rice (Oparka and Gates 1981a, b). MLG has been also reported to play an important role in cell expansion in maize coleoptiles (Carpita 1984; Carpita et al. 2001) and root cells (Kozlova et al. 2014).

Callose has been reported to be a key element of cell plate formation and cellularisation in many species, including wheat and rice (Morrison and Obrien 1976; Brown et al. 1997). However, it is typically reported to be transient and remodelled or remobilised after cellularisation is completed. Our data show that callose is strongly associated with the extending cell wall outgrowths of the syncytial stage in wheat and rice, but also present past the end of cellularisation. In particular, increased labelling of the periclinal cell walls of the aleurone cells and sub-aleurone cells was observed at 12 DAA in wheat, suggesting that callose may still be important in the division and differentiation of the aleurone cells into sub-aleurone cells, which occurs up to at least 15 DAA. (Evers 1970; Cochrane and Duffus 1981).

In addition to callose, strong labelling of the cell wall ingrowths in the syncytium was observed with the LM25 xyloglucan antibody both in wheat and barley. Xyloglucan has not been reported as a component of mature wheat endosperm, although Pellny et al. (2012) reported the presence of transcripts for xyloglucan synthase and immunodetection of xyloglucan in the developing endosperm. Our results show a transient detection of xyloglucan in the cellularising endosperm cell walls, similarly to that reported in barley (Wilson et al. 2012), albeit with a different xyloglucan antibody (LM15), suggesting that this may be a conserved mechanism amongst grasses marking the transition from a syncytial state to the cellularised endosperm. Xyloglucan may regulate the deposition of callose, in the same way as it is proposed to assist the deposition of cellulose fibrils (Zhou et al. 2007). It is well established that wheat endosperm cell walls contain glucomannan (Mares and Stone 1973; Pellny et al. 2012) which appears to be a clear distinction from rice endosperm where glucomannan has not been observed by biochemical analysis or immunodetection, but for which mannose has been reported by monosaccharide analysis (Lai et al. 2007). As we learn more about the specific cellular roles of matrix polymers, the basis of the differences in their deposition between the two species may become apparent.

### Pectic polysaccharides and grain development

The pectic set of polysaccharides, perhaps best viewed as sets of supramolecules with varying structurally modulated domains such as HG and RG-I, is potentially even more structurally complex and diverse (Caffall and Mohnen 2009; Burton et al. 2010) than the sets of hemicelluloses discussed above. The detection of pectic polysaccharides in the cell walls of the rice endosperm is an intriguing observation. HG was detected in the endosperm of rice at 28 DAA but only with the LM19 antibody, which binds to unesterified HG (Verhertbruggen et al. 2009). This is in contrast to the maternal tissues that were also labelled with the JIM7 antibody, which recognises HG methylesters (Clausen et al. 2003). These are interesting observations from the perspective of pectic HG biosynthesis and deposition as HG is thought to be synthesised in a methyl-esterified form and then subject to enzymic de-esterification *in muro* (Atmodjo et al. 2013). Recent work by Chateigner-Boutin et al. (2014) has reported the unmasking (by enzymic removal of abundant polysaccharides) of the LM20 high methylester HG epitope in wheat endosperm cell walls indicating that pectic HG is indeed present in wheat endosperm cells, as previously suggested by the abundance of wheat endosperm transcripts for GAUT genes that encode enzymes synthesising the HG backbone (Pellny et al. 2012).

The RG-I domains of pectic supramolecules are highly heterogeneous. Epitopes indicative of RG-I side chains (LM5 galactan and LM6 arabinan) were detected during grain development of both wheat and rice before any detection of the RG-I backbone epitope. This suggests that RG-I is present, but that either the backbone epitope is masked in some way early in development, or that structurally distinct forms of side chain polysaccharides without the acidic backbone occur. Analysis by Chateigner-Boutin et al. (2014) demonstrated that the INRA-RU1 epitope could be detected in wheat from approximately 11 DAA using enzymatic digestion of cell wall. Either scenario is indicative of dynamic changes to RG-I polymers during grain development and current views of RG-I indicate roles in the generation of cell wall mechanical properties (Caffall and Mohnen 2009). The LM5 galactan epitope was detected only in rice endosperm, from cellularisation, and by 8–20 DAA was observed to be restricted to the aleurone, sub-aleurone and two or three cell layers of endosperm cells adjacent to the sub-aleurone. The labelled cells are those exhibiting the highest rates of cell expansion, supporting a role for galactan side chains in cell elongation, which has previously been reported in the Arabidopsis root (McCartney et al. 2003). By contrast to the galactan epitope, the LM6 arabinan epitope was detected in the endosperm of both species. However, in wheat LM6 was only observed to label the endosperm cell walls from cellularisation up to 8 DAA, again coinciding with the phase of rapid cell expansion, whilst in rice endosperm labelling with LM6 was observed at all stages (8–28 DAA). The arabinan side chain of pectin has been implicated in drought resistance in resurrection plants (Moore et al. 2008) via regulation of the hydration state and water retention capacity of cell walls. It could play a similar role in the expanding wheat and rice endosperm, maintaining wall flexibility to allow for cellular expansion and flexibility. After 20 DAA, the LM6 epitope was detected at the inner face of the cell wall/internal cell organelles (Insert in Fig. 8l). The LM6 epitope can be present on arabinogalactan-proteins (AGPs) (Lee et al. 2005) and the presence of the LM6 epitope at these locations may, therefore, be indicative of AGPs rather than RG-I.

## Conclusion

The high level of sensitivity provided by mAbs allows the determination of the developmental dynamics of minor cell wall components which are likely to provide important modifications to the structures and properties of cell walls. The near synchronous deposition of AX, MLG and callose in analogous cellular locations in wheat and rice, implicates these polymers in specific developmental stages, namely cellularisation for callose, and cell differentiation for AX and MLG. Xyloglucan can also now be included as a cell wall component at cellularisation. In comparative terms, glucomannan is a distinctive feature of wheat endosperm cell walls whilst pectic galactan and glucuronoxylan appear distinctive of rice endosperm cell walls. Pectic polysaccharides, notably those of RG-I, may play important roles in maintaining cell wall integrity during the rapid cell expansion in the grain after the termination of cellularisation.

## Electronic supplementary material

Below is the link to the electronic supplementary material.

Supplementary Fig. S1. Indirect immunofluorescence detection of AX, callose and MLG in medial transverse sections of a wheat grain at 4 (**a**–**c**), 12 (**d**–**f**), and 28 DAA (**g**–**i**). Immunofluorescence detection of AX (**a**, **d**, **g**) and callose (**b**, **e**, **h**) and MLG (**c**, **f**, **i**). Al = aleurone, M = maternal pericarp, NP = nucellar projection, SE = starchy endosperm, VB = vascular bundle. Arrowheads indicate labelling of anticlinal cell wall extensions Bars = 100 μm, except D, G = 200 μm (JPEG 108 kb)

## Abbreviations

AX: Arabinoxylan
DAA: Days after anthesis
FT-IR: Fourier transform infrared spectroscopy
GalA: Galacturonic acid
GUX: Glucuronoxylan
HG: Homogalacturonan
MLG: Mixed-linkage glucan
NP: Nucellar projection
PBS: Phosphate buffer saline
RG-I: Rhamnogalacturonan-I
RG-II: Rhamnogalacturonan-II
RT: Room temperature
SE: Starchy endosperm
TS: Transverse section
VB: Vascular bundle
