# Supplementary figures and images for: Comparative in situ analyses of cell wall matrix polysaccharide dynamics in developing rice and wheat grain
Source: Planta. 2014 Nov 22;241(3):669–85. doi: 10.1007/s00425-014-2201-4 (PMC4328131; doi:10.1007/s00425-014-2201-4)

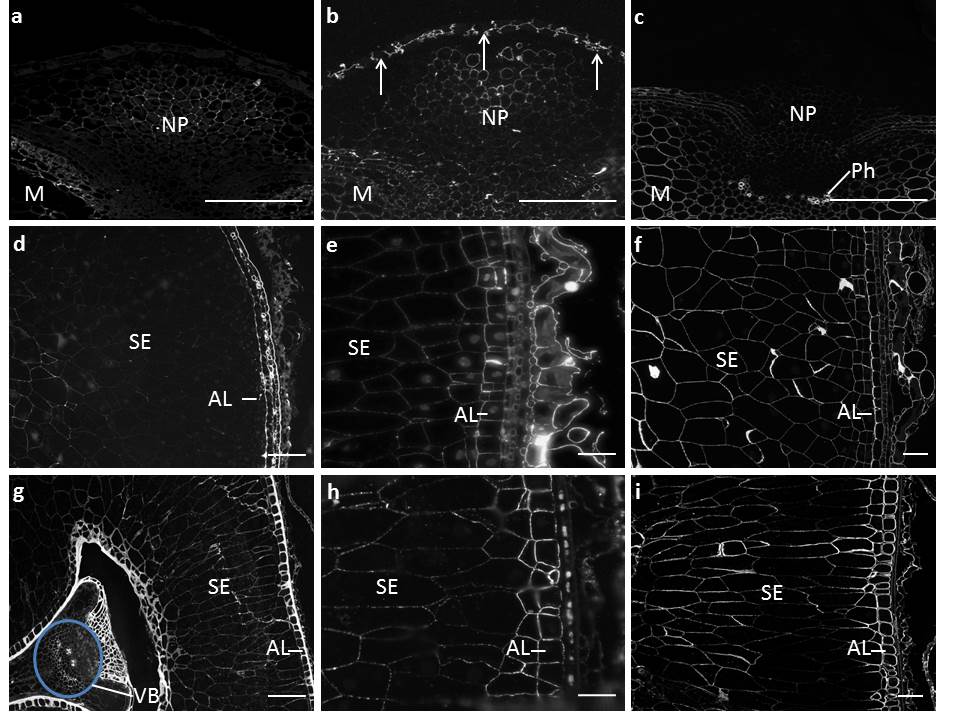

Supplement: Supplementary file 1 — Supplementary Fig. S1. Indirect immunofluorescence detection of AX, callose and MLG in medial transverse sections of a wheat grain at 4 (a–c), 12 (d–f), and 28 DAA (g–i). Immunofluorescence detection of AX (a, d, g) and callose (b, e, h) and MLG (c, f, i). Al = aleurone, M = maternal pericarp, NP = nucellar projection, SE = starchy endosperm, VB = vascular bundle. Arrowheads indicate labelling of anticlinal cell wall extensions Bars = 100 μm, except D, G = 200 μm (JPEG 108 kb) [file 425_2014_2201_MOESM1_ESM.jpg]
